# The unexpected versatility of ALP/Enigma family proteins

**DOI:** 10.3389/fcell.2022.963608

**Published:** 2022-12-01

**Authors:** Lucas A. B. Fisher, Frieder Schöck

**Affiliations:** Department of Biology, McGill University, Montreal, QC, Canada

**Keywords:** ALP/Enigma, PDLIM, PDZ, LIM, ZM, muscle, sarcomere, ZASP

## Abstract

One of the most intriguing features of multicellular animals is their ability to move. On a cellular level, this is accomplished by the rearrangement and reorganization of the cytoskeleton, a dynamic network of filamentous proteins which provides stability and structure in a stationary context, but also facilitates directed movement by contracting. The ALP/Enigma family proteins are a diverse group of docking proteins found in numerous cellular milieus and facilitate these processes among others. In vertebrates, they are characterized by having a PDZ domain in combination with one or three LIM domains. The family is comprised of CLP-36 (PDLIM1), Mystique (PDLIM2), ALP (PDLIM3), RIL (PDLIM4), ENH (PDLIM5), ZASP (PDLIM6), and Enigma (PDLIM7). In this review, we will outline the evolution and function of their protein domains which confers their versatility. Additionally, we highlight their role in different cellular environments, focusing specifically on recent advances in muscle research using *Drosophila* as a model organism. Finally, we show the relevance of this protein family to human myopathies and the development of muscle-related diseases.

## Introduction

Animal locomotion is one of the most fascinating mechanisms in nature. Evading an enemy, foraging for food, finding a mate; these fundamental processes of life all require locomotory abilities. On a cellular level, this is accomplished by the rearrangement and reorganization of the cytoskeleton, a dynamic network of filamentous proteins which provides stability and structure in a stationary context, but also facilitates directed movement by contracting. All animal cells require regulation of the cytoskeleton and their dynamics. This regulation is facilitated by numerous different classes of proteins, such as crosslinkers, kinases, docking proteins and more.

**FIGURE 1:**
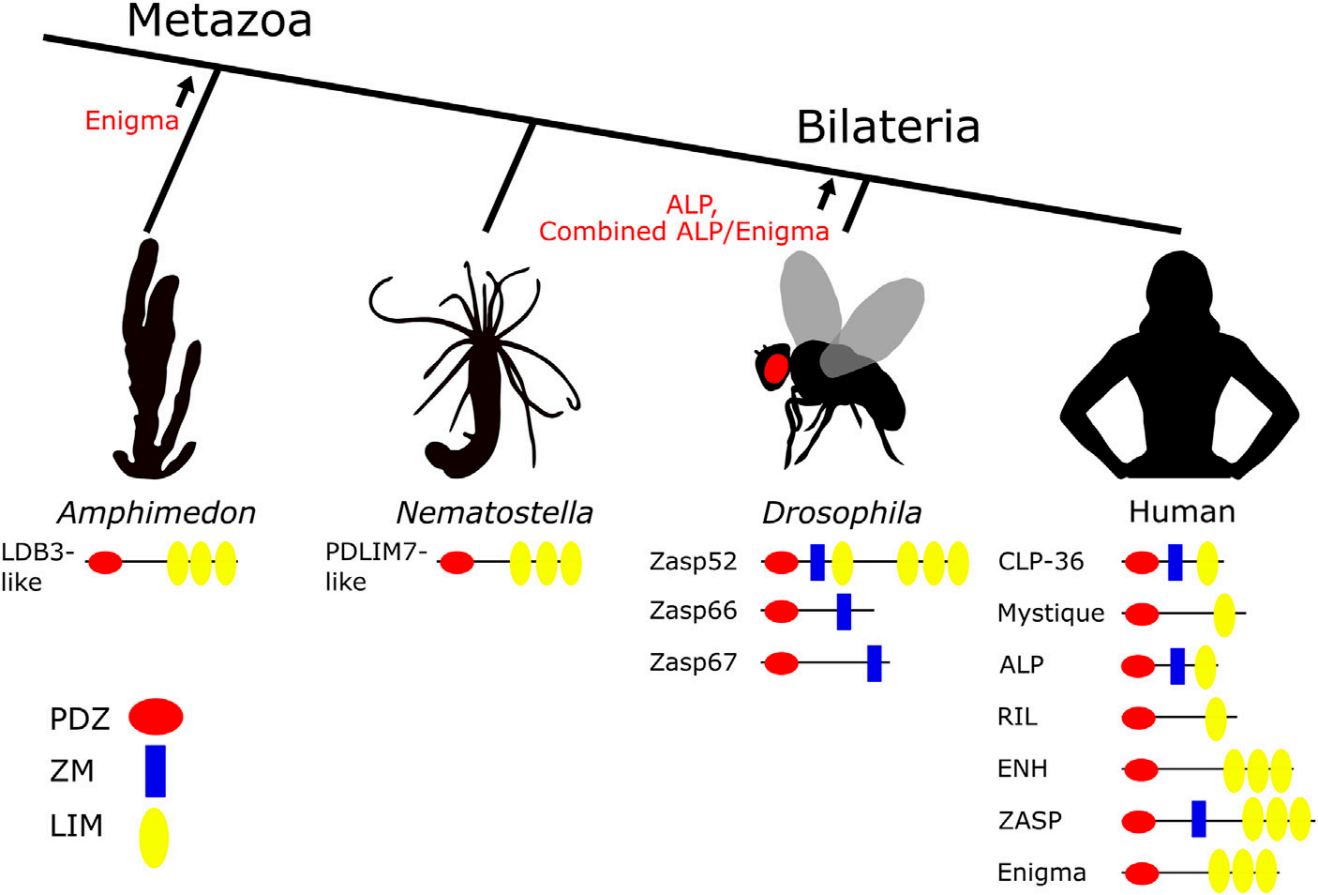
Origin and distribution of ALP/Enigma proteins in the metazoan tree. ALP/Enigma proteins first evolved in metazoans as evidenced by single Enigma-like proteins found for example in the ancient phyla of Porifera (the sponge *Amphimedon*) and Cnidaria (the sea anemone *Nematostella*). In Bilateria, duplication and recombination gave rise to ALP-like proteins with a single, but different LIM domain, but also to combined ALP/Enigma proteins with four LIM domains (e.g. in *Drosophila* and *C*. *elegans*), as well as proteins without LIM domains (Zasp66 and Zasp67 in *Drosophila*). The ZM domain first evolved in the ancestor of Bilateria. Evolutionary tree with representative species and the ALP/Enigma proteins with their respective proteins domains (PDZ, ZM, LIM) are shown. Arrows indicate the stem lineage where a particular group of ALP/Enigma proteins originated.

The ALP/Enigma family proteins are able to bind to both cytoskeletal and nuclear proteins and interact with a number of regulatory proteins (reviewed in Krcmery et al., 2010). The family is characterized by having a PDZ (post synaptic density protein, *Drosophila* discs large, and zonula occludens-1 protein) domain, LIM (Lin11, Isl-1, Mec-3) domains, a ZM (Zasp motif) domain, and an AM (ALP motif) domain (Koch et al., 2012). While the evolution of PDZ and LIM domains predates metazoans, Enigma proteins with a combination of one N-terminal PDZ domain, an AM domain, and three C-terminal LIM domains originated in the stem lineage of Metazoa, and later gave rise through duplication and recombination to both the ALP and combined ALP/Enigma subclasses in the stem of Bilateria (Figure 1). ALP proteins have a PDZ domain followed by a single, but different LIM domain. Combined ALP/Enigma proteins evolved in certain groups of Bilateria like Ecdysozoa and consist of one PDZ domain followed by one ALP-like LIM domain and three C-terminal Enigma-like LIM domains (te Velthuis et al., 2007; Koch et al., 2012) (Figure 1). *Drosophila* also has family members that have lost LIM domains entirely (González-Morales et al., 2019a) (Figure 1). Finally, the ZM domain is only found in Bilateria (González-Morales et al., 2019b). Interestingly, all members of the family share a portion of the AM domain, first described in te Velthuis et al., 2007. It was previously believed that this motif only belonged to the ALP protein subfamily, but it has now been discovered to be present in all members of the ALP/Enigma family (Koch et al., 2012). This, together with the order of domains, reinforces their familial evolutionary relationship and indicates that ALP/Enigma members shared a metazoan common ancestor.

In humans, the ALP/Enigma family is comprised of CLP-36 (PDLIM1), Mystique (PDLIM2), ALP (PDLIM3), RIL (PDLIM4), ENH (PDLIM5), ZASP (PDLIM6), and Enigma (PDLIM7) (Figure 1). The first four members belong to the ALP subfamily, while the last three members belong to the Enigma subfamily.

Here we will provide an update of the current research on proteins of the ALP/Enigma family, focusing on their cytoskeletal functions in *Drosophila* and vertebrates.

## The structure and function ofALP/Enigma family protein domains

ALP/Enigma proteins have developed a wide range of functions in the cell, and are able to bind a myriad of different proteins. This is accomplished by the combination of PDZ and LIM protein domains which on one hand can facilitate interaction with the cytoskeleton either directly or indirectly through the help of other proteins such as *a*-actinin, while also being able to interact with different cytoskeletal regulatory proteins (Table 1). Some members of this family also contain a muscle-specific ZM domain. Although not much is known of this domain, recent research has uncovered its importance in muscle development and structure, which will be a focus of discussion later in this review. The AM domain is not further discussed here because too little is known about it at the present time (Koch et al., 2012). Examining the function of these domains gives insight into how ALP/Enigma proteins developed their versatility with regards to cytoskeletal dynamics.

**TABLE 1:** Interaction partners of the ALP/Enigma family proteins.

Protein	Interactor	Domain/region	System	Method	Binding	References/date
CLP-36 (PDLIM1)	Clik1	LIM	Yeast/Tissue culture	Yeast two-hybrid/Co-immunoprecipitation	Indirect	Vallenius and Mäkelä (2002)
	EGFR	Full-length	Tissue culture	Co-immunoprecipitation	Indirect	Naegle et al. (2012)
	FATZ family	Full-length	Bacterial	AlphaScreen	Direct	von Nandelstadh et al. (2009)
	FHL1	Full-length	Mouse	Co-immunoprecipitation	Indirect	Sharma et al. (2011)
	Gelsolin	Full-length	Mouse	Co-immunoprecipitation	Indirect	Sharma et al. (2011)
	Glycoprotein VI	Full-length	Mouse	Co-immunoprecipitation	Indirect	Gupta et al. (2012)
	Linker of activated T cells (LAT)	Full-length	Mouse	Co-immunoprecipitation	Indirect	Gupta et al. (2012)
	Myotilin	Full-length	Bacterial	AlphaScreen	Direct	von Nandelstadh et al. (2009)
	Palladin	PDZ	Yeast/Tissue culture/Mouse	Yeast two-hybrid/Co-immunoprecipitation	Indirect	Hasegawa et al. (2010)
			Tissue culture	Pull-down assay/Co-immunoprecipitation	Direct	Maeda et al. (2009)
	Plasma membrane Ca^2+^-ATPase	Full-length	Human platelet	Co-immunoprecipitation/Pull-down assay	Indirect	Bozulic et al. (2007)
	p75^NTR^	PDZ	Bacterial/Human	Pull-down assay/Co-immunoprecipitation	Direct	Ahn et al. (2015)
	SHC1	Full-length	Tissue culture	Co-immunoprecipitation	Indirect	Naegle et al. (2012)
	STIM1	Full-length	Mouse	Co-immunoprecipitation	Indirect	Gupta et al. (2012)
	α-actinin	Full-length	Tissue culture	Co-immunoprecipitation	Indirect	Liu Z et al. (2014)
			Tissue culture	Co-immunoprecipitation	Indirect	Naegle et al. (2012)
			Mouse	Co-immunoprecipitation	Indirect	Sharma et al. (2011)
			Bacterial	AlphaScreen	Direct	von Nandelstadh et al. (2009)
			Human platelet	Co-immunoprecipitation	Indirect	Bozulic et al. (2007)
		Interdomain region	Tissue culture	Pull-down assay	Direct	Maeda et al. (2009)
			Human platelet/Yeast	Co-immunoprecipitation/Yeast two-hybrid	Indirect	Bauer et al. (2000)
		LIM	Yeast/Rat	Yeast two-hybrid/Co-immunoprecipitation	Indirect	Kotaka et al. (2000)
		PDZ	Tissue culture	Co-immunoprecipitation	Indirect	Ono et al. (2015)
			Human kidney tissue culture	Co-immunoprecipitation	Indirect	Liu et al. (2011)
			Tissue culture	Pull-down assay	Direct	Maeda et al. (2009)
			Bacterial	Surface plasmon resonance	Direct	Klaavuniemi et al. (2004)
			Bacterial/Tissue culture	Pull-down assay/Co-immunoprecipitation	Direct	Vallenius et al. (2000)
	β-catenin/E-cadherin complex	PDZ	Tissue culture	Co-immunoprecipitation	Indirect	Chen et al. (2015)
Mystique (PDLIM2)	Actin	Extended PDZ domain	Bacterial	Pull-down assay	Direct	Liao et al. (2020)
	Amotl1	Full-length	Tissue culture	Co-immunoprecipitation	Indirect	Sistani et al. (2011)
	COP9 Signalosome	Full-length	Tissue culture	Co-immunoprecipitation/peptide array	Indirect	Bowe et al. (2014)
	Enigma (PDLIM7)	LIM	Tissue culture	Co-immunoprecipitation	Indirect	Jodo et al. (2020)
	Filamin A	Full-length	Rat	Co-immunoprecipitation/Overlay assay	Indirect	Torrado et al. (2004)
	HN12-NS1	PDZ	Yeast/Bacterial/Tissue Culture	Yeast two-hybrid/Pull-down assay/Mammalian two-hybrid/Bimolecular fluorescence complementation	Direct	Yu et al. (2011)
	HSP70	Full-length	Tissue culture	Co-immunoprecipitation	Indirect	Tanaka et al. (2014)
	HSP90	Full-length	Tissue culture	Co-immunoprecipitation	Indirect	Tanaka et al. (2014)
	Hsc70	Full-length	Tissue culture	Co-immunoprecipitation	Indirect	Tanaka et al. (2014)
	IκB-ζ	LIM	Tissue culture	Co-immunoprecipitation	Indirect	Kimura et al. (2018)
	Myosin heavy chain IIA	Full-length	Rat	Co-immunoprecipitation	Indirect	Torrado et al. (2004)
	Myosin VI	Full-length	Rat	Co-immunoprecipitation	Indirect	Torrado et al. (2004)
	NQO1	LIM	Tissue culture	Co-immunoprecipitation	Indirect	Kimura et al. (2018)
	OspE1	PDZ	Bacterial	Pull-down assay	Direct	Yi et al. (2014)
	Palladin	Full-length	Yeast	Yeast two-hybrid	Indirect	Hasegawa et al. (2010)
			Tissue culture	Pull-down assay	Direct	Maeda et al. (2009)
	p65	Full-length	Tissue culture	Co-immunoprecipitation	Indirect	Tanaka et al. (2007)
	STAT2	Full-length	Tissue culture	Co-immunoprecipitation	Indirect	Joyce et al. (2019)
	STAT3	LIM	Tissue culture	Co-immunoprecipitation	Indirect	Tanaka et al. (2011)
	Tax	Interdomain region	Bacterial	Co-immunoprecipitation	Direct	Fu et al. (2010)
	α-actinin	Full-length	Tissue culture	Co-immunoprecipitation	Indirect	Sistani et al. (2011)
			Rat/Bacterial	Co-immunoprecipitation/Pull-down assay	Direct	Torrado et al. (2004)
ALP (PDLIM3)	Actin	Full-length	Bacterial	Co-sedimentation assay	Direct	Pashmforoush et al. (2001)
	FATZ family	Full-length	Bacterial	AlphaScreen	Direct	von Nandelstadh et al. (2009)
	LET-502	Full-length	Tissue culture	Yeast two-hybrid/Co-immunoprecipitation	Indirect	Shimizu et al. (2018)
	Myotilin	Full-length	Bacterial	AlphaScreen	Direct	von Nandelstadh et al. (2009)
	RIL (PDLIM4)	Full-length	Tissue culture	Co-immunoprecipitation	Indirect	van den Berk et al. (2005)
	α-actinin	Full-length	Bacterial	AlphaScreen	Direct	von Nandelstadh et al. (2009)
			Bacterial	Surface plasmon resonance	Direct	Klaavuniemi and Ylanne 2006
			Chicken/Tissue culture	Overlay assay/Solid-phase binding assay/Co-immunoprecipitation	Direct	Pomiès et al. (1999)
		Interdomain region	Bacterial	Surface plasmon resonance/peptide binding study	Direct	Klaavuniemi et al. (2009)
		PDZ	Bacterial	Surface plasmon resonance	Direct	Klaavuniemi et al. (2004)
			Bacterial	Overlay assay	Direct	Henderson et al. (2003)
			Yeast/Bacterial/Tissue culture	Yeast two-hybrid/Pull-down assay/Co-immunoprecipitation	Direct	Xia et al. (1997)
		ZM	Bacterial	Surface plasmon resonance	Direct	Klaavuniemi et al. (2004)
RIL (PDLIM4)	Actin	Full-length	Bacterial	Co-sedimentation assay	Direct	Vanaja et al. (2009)
		LIM	Mouse	Co-immunoprecipitation	Indirect	Fu et al. (2019)
	ALP (PDLIM3)	LIM	Tissue culture	Co-immunoprecipitation	Indirect	van den Berk et al. (2005)
	GluR-A	LIM and extended region	Yeast/Tissue culture	Yeast two-hybrid/Co-immunoprecipitation/Pull-down assay	Direct	Schulz et al. (2004)
	Palladin	Full-length	Yeast	Yeast two-hybrid	Indirect	Hasegawa et al. (2010)
			Tissue culture	Pull-down	Direct	Maeda et al. (2009)
	PTP-BL	C-terminus	Bacterial	Equilibrium and Kinetic binding experiments	Direct	Toto et al. (2017)
			Tissue culture/Yeast	Pull-down assay/Nuclear Magnetic Resonance/Yeast two-hybrid	Direct	van den Berk et al. (2005)
			Bacterial	Nuclear Magnetic Resonance	Direct	Walma et al. (2002)
		LIM (plus C-terminus extension)	Tissue culture/Yeast	Co-immunoprecipitation/Yeast two-hybrid	Indirect	Cuppen et al. (1998)
	Self-interaction	PDZ/LIM	Tissue culture/Yeast	Yeast two-hybrid/Co-immunoprecipitation	Indirect	Cuppen et al. (1998)
	Sphingosine-1-phosphate receptor 1	PDZ	Mouse	Co-immunoprecipitation	Indirect	Fu et al. (2019)
	TRIP6	PDZ	Yeast/Tissue culture	Yeast two-hybrid/Co-immunoprecipitation	Indirect	Cuppen et al. (2000)
	α-actinin	PDZ	Yeast/Tissue culture	Yeast two-hybrid/Pull-down assay/Co-immunoprecipitation	Direct	Schulz et al. (2004)
			Tissue culture/Bacterial	Co-immunoprecipitation/Overlay assay	Direct	Vallenius et al. (2004)
ENH (PDLIM5)	Actin	PDZ	Tissue culture/Bacterial	Pull-down assay	Direct	Nakagawa et al. (2000)
	AE1C	PDZ	Bacterial/Tissue culture	ELISA assay/Pull-down assay	Direct	Su et al. (2017)
	AKT1	LIM	Tissue culture	Co-immunoprecipitation	Indirect	Huang et al. (2020)
	AMPK	Full-length	Tissue culture	Co-immunoprecipitation	Indirect	Liu et al. (2017)
		LIM	Tissue culture	Co-immunoprecipitation	Indirect	Yan et al. (2015)
	Calsarcin	Full-length	Mouse/Tissue culture	Co-sedimentation assay/Co-immunoprecipitation	Indirect	Cheng et al. (2010)
	CREB	LIM (third LIM domain)	Rat/Tissue culture	Co-immunoprecipitation	Indirect	Ito et al. (2015)
	Id2	Full-length	Tissue culture	Co-immunoprecipitation	Indirect	Nakatani et al. (2016)
		LIM	Yeast/Bacterial/Tissue culture	Yeast two-hybrid/Pull-down assay/Co-immunoprecipitation	Direct	Lasorella and Iavarone (2006)
	ILK	LIM	Bacterial/Tissue culture	Pull-down assay/Overlay assay	Direct	Su et al. (2017)
	L-type Ca^2+^ channel	PDZ	Tissue culture	Co-immunoprecipitation	Indirect	Maturana et al. (2008)
	Myotilin	Full-length	Mouse/Tissue culture	Co-immunoprecipitation	Indirect	Cheng et al. (2010)
	N-type Ca^2+^ channel	Full-length	Rat	Co-immunoprecipitation	Indirect	Maeno-Hikichi et al. (2003)
		LIM (second LIM domain) and linker region	Bacterial/Tissue culture	Pull-down assay/Co-immunoprecipitation	Direct	Chen et al. (2006)
	PHLPP1 and 2	PDZ	Tissue culture	Co-immunoprecipitation	Indirect	Huang et al. (2020)
	Protein Kinase A	Full-length	Tissue culture	Co-immunoprecipitation	Indirect	Lin et al. (2013)
	Protein Kinase C	Full-length	Rat	Co-immunoprecipitation	Indirect	Ren et al. (2015)
			Rat	Co-immunoprecipitation	Indirect	Maeno-Hikichi et al. (2003)
		LIM	Tissue culture	Co-immunoprecipitation	Indirect	Maturana et al. (2011)
		LIM (second LIM domain)	Bacterial/Tissue culture	Pull-down assay/Co-immunoprecipitation	Direct	Chen et al. (2006)
		LIM	Yeast/Tissue culture	Yeast two-hybrid/Co-immunoprecipitation	Indirect	Kuroda et al. (1996)
	Protein Kinase D	LIM (second LIM domain)	Yeast/Tissue culture	Yeast two-hybrid/Co-immunoprecipitation	Indirect	Maturana et al. (2008)
	Self-interaction	Full-length	Tissue culture	Co-immunoprecipitation	Indirect	Chen et al. (2006)
	SMAD3	LIM	Tissue culture	Co-immunoprecipitation	Indirect	Shi et al. (2020)
	SPAR	Full-length	Yeast/Rat	Yeast two-hybrid/Co-immunoprecipitation	Indirect	Herrick et al. (2010)
	YAP	Full-length	Tissue culture	Co-immunoprecipitation	Indirect	Elbediwy et al. (2018)
	ZASP (PDLIM6)	Full-length	Mouse	Co-sedimentation assay	Indirect	Cheng et al. (2010)
	α-actinin	Full-length	Tissue culture	Co-immunoprecipitation	Indirect	Elbediwy et al. (2018)
			Tissue culture	Co-immunoprecipitation	Indirect	Ren et al. (2015)
			Mouse	Overlay assay	Direct	Niederländer et al. (2004)
		PDZ	Bacterial/Tissue culture	Pull-down assay	Direct	Nakagawa et al. (2000)
	δ-catenin	PDZ	Bacterial	Protein-domain microarray	Direct	Baumert et al. (2020)
ZASP (PDLIM6)	Actin	Extended PDZ domain	Bacterial	Pull-down assay/Surface plasmon resonance	Direct	Liao et al. (2020)
		Various regions	Bacterial	Surface plasmon resonance/Nuclear magnetic resonance	Direct	Watts et al. (2017)
		ZM	Yeast/Mouse/Tissue culture	Yeast two-hybrid/Co-immunoprecipitation/Pull-down assay/Slot blot assay	Direct	Lin et al. (2014)
	Ankrd2	PDZ and ZM	Tissue culture/Bacterial	Co-immunoprecipitation/Overlay assay	Direct	Martinelli et al. (2014)
	Calsarcin	Full-length	Mouse	Co-sedimentation assay	Indirect	Cheng et al. (2010)
			Yeast/Tissue culture	Yeast two-hybrid/Co-immunoprecipitation	Indirect	Frey and Olson (2002)
		PDZ	Yeast/Mouse	Yeast two-hybrid/Co-immunoprecipitation	Indirect	Zheng et al. (2008)
	Ca_v_1,2 (L type calcium channel)	Full-length	Bacterial/Rat	Pull-down assay	Direct	Li et al. (2010)
	ENH (PDLIM5)	Full-length	Mouse	Co-sedimentation assay	Indirect	Cheng et al. (2010)
	FATZ family	PDZ	Bacterial	Pull-down assay/AlphaScreen	Direct	von Nandelstadh et al. (2009)
	Filamin C	Exons 8–11Δ10	Yeast/Tissue culture/Mouse	Yeast two-hybrid/Co-immunoprecipitation/Pull-down assay	Direct	Pathak et al. (2021)
	HSPA8	Exons 8–11Δ10	Yeast/Tissue culture/Mouse	Yeast two-hybrid/Co-immunoprecipitation	Indirect	Pathak et al. (2021)
	Myotilin	PDZ	Bacterial/Tissue culture	Yeast two-hybrid/*In vitro* pull-down/Co-immunoprecipitation	Direct	von Nandelstadh et al. (2009)
			Yeast/Mouse	Yeast two-hybrid/Co-immunoprecipitation	Indirect	Zheng et al. (2008)
	Na_v_1.5 (Sodium channel)	Full-length	Bacterial/Rat	Pull-down assay	Direct	Li et al. (2010)
	Phosphoglucomutase 1	ZM and extended region (exon 4, 6, 10)	Tissue culture	Co-immunoprecipitation	Indirect	Arimura et al. (2009)
	Protein Kinase A	Cypher Cardiac Specific Region	Tissue culture/Rat	Co-immunoprecipitation	Indirect	Lin et al. (2013)
	Protein Kinase C	Full-length	Tissue culture	Co-immunoprecipitation	Indirect	Yamashita et al. (2014)
		LIM	Tissue culture	Yeast two-hybrid/Pull-down assay	Direct	Arimura et al. (2004)
			Tissue culture	Co-immunoprecipitation	Indirect	Zhou et al. (1999)
	p53	PDZ	Tissue culture	Co-immunoprecipitation/Overlay assay	Direct	Martinelli et al. (2014)
	Self-interaction	LIM/ZM	Yeast/Fly	Yeast two-hybrid/Bimolecular fluorescence complementation	Indirect	González-Morales et al. (2019a)
		LIM (third LIM domain)	Yeast	Yeast two-hybrid	Indirect	Arimura et al. (2004)
	ZO-2	PDZ	Tissue culture/Bacterial	Co-immunoprecipitation/Pull-down assay	Direct	Lechuga et al. (2010)
	α-actinin	Full-length	Yeast	Yeast two-hybrid	Indirect	Seto et al. (2011)
			Bacterial	Surface plasmon resonance	Direct	Klaavuniemi and Ylanne (2006)
		Extended PDZ domain	Fly/Bacterial	Co-immunoprecipitation/Pull-down assay	Direct	Liao et al. (2016)
		PDZ	Yeast	Yeast two-hybrid	Indirect	Lin et al. (2014)
			Bacterial	Nuclear magnetic resonance	Direct	Au et al. (2004)
			Tissue culture	Co-immunoprecipitation	Indirect	Zhou et al. (1999)
		ZM	Tissue culture	Co-immunoprecipitation	Indirect	Martinelli et al. (2014)
Enigma (PDLIM7)	Actin	Extended PDZ domain	Bacterial	Pull-down assay	Direct	Liao et al. (2020)
	APS	LIM (third LIM domain)	Yeast/Tissue culture	Yeast two-hybrid/Co-immunoprecipitation	Indirect	Barrès et al. (2005)
		Full-length	Tissue culture	Co-immunoprecipitation	Indirect	Barrès et al. (2006)
	Cbl-c	LIM (first LIM domain)	Yeast/Tissue culture	Yeast two-hybrid/Pull-down assay/Co-immunoprecipitation	Direct	Kales et al. (2014)
	Insulin-like growth factor binding protein	Full-length	Yeast/Tissue culture	Yeast two-hybrid/Co-immunoprecipitation	Indirect	Strohbach et al. (2008)
	Insulin receptor *ß*	LIM (second LIM domain)	Yeast	Yeast two-hybrid	Indirect	Wu and Gill (1994)
	Jab1	Interdomain region	Tissue culture	Biotin transfer assay/Slot blot assay/Co-immunoprecipitation	Direct	Sangadala et al. (2013)
	MDM2	Full-length	Tissue culture	Co-immunoprecipitation	Indirect	Klein et al. (2018)
		LIM (third LIM domain)	Tissue culture/Bacterial	Co-immunoprecipitation/Pull-down	Direct	Jung et al. (2010)
	Mystique (PDLIM2)	LIM (second LIM domain)	Tissue culture	Co-immunoprecipitation	Indirect	Jodo et al. (2020)
	Nedd4-1	Interdomain region	Bacterial	Pull-down assay	Direct	D’Cruz et al. (2016)
	OspE1	PDZ	Tissue culture/Bacterial	Co-immunoprecipitation/Pull-down assay	Direct	Yi et al. (2014)
	Protein Kinase C	LIM	Tissue culture	Pull-down assay	Direct	Kuroda et al. (1996)
	p65	Full-length	Tissue culture	Co-immunoprecipitation	Indirect	Jodo et al. (2020)
	RET	Full-length	Tissue culture	Co-immunoprecipitation	Indirect	Borrello et al. (2002)
		LIM (second LIM domain)	Tissue culture	Co-immunoprecipitation	Indirect	Kales et al. (2014)
			Tissue culture	Co-immunoprecipitation	Indirect	Durick et al. (1998)
			Yeast/Tissue culture/Bacterial	Yeast two-hybrid/Pull-down assay	Direct	Durick et al. (1996)
	Smurf1	Interdomain region	Bacterial	Ligand blot assay/Slot blot assay	Direct	Sangadala et al. (2007)
			Bacterial/Tissue culture	Biotin transfer assay/Co-immunoprecipitation/Slot blot assay	Direct	Sangadala et al. (2006)
	YAP	Full-length	Tissue culture	Co-immunoprecipitation	Indirect	Elbediwy et al. (2018)
	α-actinin	Full-length	Tissue culture	Co-immunoprecipitation	Indirect	Elbediwy et al. (2018)
	β-tropomyosin	PDZ	Bacterial/Tissue culture	Pull-down assay/Co-immunoprecipitation	Direct	Guy et al. (1999)

### The PDZ domain and the key to versatility

The PDZ domain is a common structural domain found in most organisms, and one of the most prevalent interaction modules found in humans (reviewed in Harris and Lim 2001 and Luck et al., 2012). It is composed of six *β*-strands, capped by one long and one short *α*-helix (reviewed in Lee and Zheng 2010). Classically, PDZ domains have been organized into three unique classes based on their C-terminal binding motif; class I, which bind a S/T-X-Φ motif (where Φ is a hydrophobic amino acid and X is any amino acid), class II which bind a Φ-X-Φ motif, and class III which recognize D/E-X-Φ. There are also non-canonical PDZ motifs that can recognize internal sequences of target proteins, as well as phospholipids (Lenfant et al., 2010; reviewed in Gallardo et al., 2010). However, this three-pronged, narrow classification has been shown to be simplistic, and in reality, the PDZ binding specificity is often mediated by more than just the terminal 3-4 residues. Recent studies have shown that most PDZ domain binding sites are achieved through interactions with residues comprising the last seven amino acids, and that the PDZ domains can actually be grouped into 16 distinct specificity classes (Tonikian et al., 2008).

To further complicate things, there are other factors that determine PDZ binding cleft specificity. Indirect residue interactions (Ernst et al., 2014) and sequence context (defined as extensions directly upstream or downstream of the PDZ domain) (reviewed in Wang et al., 2010 and Luck et al., 2012) can be important for interaction with ligands. This is evident in members of the ALP/Enigma family. Zasp52, the *Drosophila* homolog of ZASP, has been shown to bind *α*-actinin, and thus be a core component of the Z-disc. It was previously believed that the interaction was mediated by the PDZ domain alone (Katzemich et al., 2013). However, recent *in vitro* biochemical data has revealed that it is not only the PDZ domain, but also a 20–50 amino acid C-terminal extension that mediates this interaction (Liao et al., 2016).

PDZ domains have also been shown to be robust to mutational load during evolution. Studying PDZ domain evolution *in vitro* using peptide phage display libraries has demonstrated that model PDZ domains are often still able to bind C-terminal peptides after undergoing single point mutations (Tonikian et al., 2008). They are “hardwired” for ligand binding, and their functional properties could permit the rapid evolution of a protein interaction network (Ernst et al., 2009). Additionally, evolved synthetic domains bind their corresponding ligands with higher affinity than reference domains that were not evolved to do so, but interestingly, in comparison to unevolved synthetic domains, they do so with lower specificity (Ernst et al., 2010). This further validates the inherent ability for the PDZ domain to bind cognate partners and its robustness to mutation during evolution.

Recently, insights into the evolutionary paths of PDZ domains have been elucidated which has direct relevance for the ALP/Enigma family. Teyra et al. (2019), using peptide phage display, mapped all possible mutational transitions between the Erbin PDZ domain, a canonical type I domain, and a synthetic Erbin-PDZ variant E-14, which exhibits atypical specificity and shows strong resemblance to the PDZ domain of PDLIM4, a member of the ALP/Enigma family. It was discovered that three substitutions alone conferred two distinct binding specificities, one similar to Erbin-PDZ and the other to that of E-14/PDLIM4, and that four or more substitutions was able to completely convert the binding profile of the variant to that of E-14/PDLIM4. All other members of the ALP/Enigma family demonstrate class I canonical binding (Au et al., 2004; Kalyoncu et al., 2010) including PDLIM3, the closest evolutionary relative to PDLIM4 in regard to the PDZ domain (te Velthuis et al., 2007). Their research showcases that minimal molecular changes in a binding site can drastically modify binding specificity, and provides an understanding of how the PDZ domain of the ALP/Enigma common ancestor could have developed a non-canonical binding specificity during evolution.

This demonstrates the versatility that can be conferred by the inclusion of the PDZ domain in protein structures. The PDZ domain can not only evolve vastly different binding specificities during evolution because of its inherent functional plasticity, but even structurally similar PDZ domain sequences can bind vastly different ligands (Ernst et al., 2009) (Table 1). Moreover, sequence context can often dictate new specificities, which continues to increase the number of protein-protein interactions possible. This explains how the ALP/Enigma family of proteins are able to interact with numerous different regulatory proteins, and how they have become crucial to vastly different processes in the cell.

### The LIM domain and mechanosensitive localization to the cytoskeleton

The LIM domain is less well understood than the PDZ domain, but it also boasts an amazing diversity in binding specificities. LIM domains are cysteine and histidine-rich zinc finger protein binding interfaces with the classical consensus sequence of CX_2_CX_16-23_HX_2_CX_2_CX_2_CX_16-21_CX_2_(C/H/D) (where X denotes any amino acid) and are roughly 50–65 amino acids in length (reviewed in Kadrmas and Beckerle 2004). LIM domains are especially difficult to classify, because the invariant cysteines required to form the zinc finger motif give the false impression of high evolutionary conservation. In reality, LIM domain sequences show low sequence conservation, and are promiscuous in their binding nature (Koch et al., 2012). During their evolution, LIM domains underwent a rapid expansion and burst of promiscuity in the stem lineage of Metazoa, likely having an important contribution in the development of animal multicellularity (Koch et al., 2012).

As for the function of LIM domains, they have been found to bind cytoskeletal proteins and are prominent members of integrin adhesion sites. Integrins transduce mechanical forces from the cellular matrix into biochemical signals in cells. This is achieved *via* the adhesome, a multiprotein complex composed of cytoskeletal proteins, adaptor proteins and numerous enzymes, which are recruited to integrin adhesion sites. Integrins have no catalytic activity themselves and are incapable of binding F-actin, thus it is necessary to recruit other proteins to the adhesome in order to translate the external forces recognized by the integrin receptors. Each member of the ALP/Enigma family have been shown to be implicated in integrin adhesion sites (Schiller et al., 2011; Bouaouina et al., 2012). Interestingly, they are part of a group of proteins that appear to be recruited to these sites in a myosin-II-dependent manner, suggesting a possible role of LIM domain proteins as tension sensors (Schiller et al., 2011).

Recently, large strides have been made to further understand this recruitment process. Two studies (Sun et al., 2020; Winkelman et al., 2020) uncovered a mechanism by which LIM domains recognize and adhere to the cytoskeleton. LIM domain proteins were found to recognize stress fiber strain sites, areas of stress fibers that are undergoing mechanical stress. This mechanism is accomplished by the LIM domains themselves, and it is facilitated *via* multiple domains working in tandem separated by pre-set linker sequences of precise length acting as a “ruler.” When the linker sequences are increased, stress fiber strain site binding is abrogated (Winkelman et al., 2020). Additionally, there is an increase in affinity to the stress fiber strain sites with an increase in the number of LIM domains (Winkelman et al., 2020). Interestingly, chimera experiments demonstrated that the LIM domains are functionally swappable (Sun et al., 2020). All of this has relevance for the Enigma subfamily of proteins, which have 3 LIM domains in tandem. This mechanism is conserved in these proteins and contributes to their localization to integrin adhesion sites (Winkelman et al., 2020). However, it is important to note that there are likely other modes by which LIM domains can engage F-actin (Sun et al., 2020), which does not preclude ALP subfamily proteins from interacting with stress fibers. Sun et al. (2020) found that ALP family members (PDLIM1 and PDLIM2) were still recruited to strain sites, but were recruited at a substantially later time point, and their recruitment was associated with an accumulation of *α*-actinin. This demonstrates that although ALP subfamily members only have one LIM domain, they are still able to reach the stress fiber strain sites in another manner, perhaps mediated by their interaction with *α*-actinin.

The stress fiber strain site recognition mechanism is highly conserved, as demonstrated by its presence in the fission yeast protein paxillin-like 1 (Pxl1). Winkelman et al. (2020) hypothesize that this mechanosensing mechanism emerged *via* a duplication and divergence event of an ancestral CRP-like LIM domain. CRP-like proteins are found in plants and are able to bind unstrained actin filaments *via* tandem LIM domains (Thomas et al., 2007). The divergence of the CRP-like ancestral molecule conferred new specificity for strained actin filaments and later underwent an expansion in the metazoan stem lineage (Winkelman et al., 2020).

LIM domain proteins are critical to the ALP/Enigma proteins. They enable the proteins of the family to recognize stressed cytoskeletal areas, and interact with a slew of different proteins (Table 1). In the context of Cypher/Zasp, LIM domains bind protein kinases (PKC), kinase anchoring proteins (AKAP) and contribute to self-interaction, which will be elaborated upon later (González-Morales et al., 2019b). For members of the Enigma subfamily, having multiple LIM domains in tandem not only increases binding to the actin stress fibres, but can also be used to bind several different proteins at once, highlighting the versatility the proteins can have in regards to protein-protein interactions. In the next section we will finish up the important domains of the ALP/Enigma family with the ZM domain, a domain less well studied than the other two, but with great leaps recently made in uncovering its function.

### The muscle-specific ZM domain: The dark horse

Almost nothing is known about the ZM domain, despite many disease-causing mutations being found within this short motif. The ZM motif is found in *Drosophila* Zasp52, human ZASP, as well as the human ALP proteins PDLIM1 and PDLIM3, which indicates that this domain was likely present in the ancestor before the splitting of subfamilies (Koch et al., 2012). Whereas PDZ domains occur in all branches of life, and LIM domains occur in all eukaryotes, the more recently evolved ZM domain is restricted to Bilateria, higher eukaryotes with canonical sarcomere structure (González-Morales et al., 2019b). This suggests muscle-specific functions, and the first well-documented function of the ZM domain is sarcomere width control, which will be described in a later section.

Limited structural studies like NMR have so far not detected a structure for this 26 amino acid motif, indicating that it could be part of a low-complexity/disordered region (Klaavuniemi et al., 2009; Watts et al., 2017). It has been proposed that the ZM domain is needed for protein-protein interactions with *α*-actinin, although this interaction is ambiguous, with some studies reporting binding and others not. For example, for both ALP and ZASP there is evidence demonstrating that the ZM domain is necessary for optimal interaction with *α*-actinin (Klaavuniemi et al., 2004; Martinelli et al., 2014). This could also be the case for CLP36, which contains similar interaction sites for *α*-actinin (Klaavuniemi et al., 2004). However, other studies, such as Liao et al. (2016) and Lin et al. (2014) found that the ZM domain was not necessary for this interaction, attributing the *α*-actinin binding to the PDZ or extended PDZ domain region.

In the case of ALP, the ZM domain is necessary for proper recruitment to the Z-disc (Klaavuniemi et al., 2004), and has been proposed to cause the symptoms of myotonic dystrophy type 1 because of faulty alternative splicing events that result in the loss of this domain (Ohsawa et al., 2011). For ZASP, the ZM domain is also necessary for proper recruitment to the Z-disc. Point mutations in this region do not seem to have effects on this phenomenon. The ZM domain mutations A165V and A171T, which are involved in myopathies, have been shown to have no effect on actin binding (Watts et al., 2017), nor on proper recruitment to the Z-disc (Klaavuniemi and Ylänne 2006). However, these mutations do impair binding to Ankrd2, a mechanosensing protein involved in regulation of gene expression and muscle differentiation, as well as *α*-actinin, highlighting possible reasons for development of muscle related diseases (Martinelli et al., 2014). Additionally, the importance of the ZM domain in determining sarcomere size discussed later suggests that these mutations could be implicated in sarcomere size control and development. Further research needs to be carried out to clarify the role of ZM mutations in sarcomere structure and regulation.

We will now briefly touch on stretches of amino acids found in the ALP/Enigma family that have no known purpose at the moment, but are present in larger isoforms in the intervening sequences between conserved domains. Using ZASP as an example, we will examine what these regions mean for the proteins of this family, and briefly discuss the burgeoning research into intrinsically disordered proteins.

### ZASP and the disordered regions of the ALP/Enigma subfamily

Intrinsically disordered protein regions are found between foldable domains and do not form a stable structure, yet are still able to carry out biological functions in a cellular environment (reviewed in Oldfield and Dunker 2014). Many of the ALP/Enigma proteins harbor interactors through binding of the interdomain area, highlighting the importance of these disordered regions (Table 1). In the case of ZASP, there are numerous isoforms that contain disordered regions.

In *Drosophila*, there are 22 isoforms of Zasp52. Of the known isoforms, the largest is roughly 240 kDa in size and is found exclusively in the indirect flight muscles (IFM) of adults (Chechenova et al., 2013). It contains an enormous stretch of roughly 1700 amino acids in between the first and second LIM domain that corresponds to an intrinsically disordered region. RNAi knockdown of these long Zasp52 isoform cannot be rescued by a transgene containing only PDZ, ZM and LIM domains (Liao et al., 2016). This data suggests that the disordered region contributes to proper structure and function of the IFMs.

In mice, some ZASP isoforms also have different regions and lengths depending on their localization. For example, some isoforms of ZASP contain tissue-specific disordered regions based on their localization pattern in cardiac or skeletal muscle (Huang et al., 2003). This indicates tissue-specific importance of these disordered regions. Additionally, some of the larger isoforms contain a homologous disordered region found before the 3 LIM domains that is necessary for proper structure and function, leading to myopathic phenotypes when lost (Huang et al., 2003). Furthermore, we already know these regions to be important for human disease. There are multiple mutations found within the linker sequences between the PDZ and LIM domains causing dilated cardiomyopathy and left ventricular non-compaction (Vatta et al., 2003). All of these studies demonstrate the importance of the ZASP intrinsically disordered regions in aberrant muscle phenotypes. Further research will need to be carried out in order to elucidate the role of these non-conserved regions in muscular structure and development, and the mechanism by which they complement the PDZ, LIM and ZM domains of the ALP/Enigma proteins to carry out cellular functions.

## The many functions of the ALP/Enigma proteins

The ALP/Enigma proteins participate in a wide array of cellular processes. For one, the ALP/Enigma proteins have been shown to be implicated in many different molecular mechanisms in the nervous system including neuritogenesis (Ohno et al., 2009; Hasegawa et al., 2010) and dendrite formation (Iida et al., 2009; Herrick et al., 2010). Additionally, and perhaps in consequence of this, there have been links of multiple members of the family with neurological diseases such as bipolar disorder, schizophrenia, attention deficit/hyperactivity disorder and Alzheimer’s disease (Kato et al., 2005; Li et al., 2008; Wang et al., 2012; Lee et al., 2015; Moselhy et al., 2015) and even some experimental evidence of ENH expression being related to mood disorders in mice (Horiuchi et al., 2013; Lu et al., 2020). ALP/Enigma proteins are also associated with tumor invasiveness. Almost all of the members of the family have been shown to be involved in cancer-related regulatory dynamics (PDLIM1: Liu et al., 2014; Chen et al., 2015; PDLIM2: Qu et al., 2010; Bowe et al., 2014; PDLIM3: Stein et al., 2010; Katkoori et al., 2012; PDLIM4: Vanaja et al., 2009; Jia et al., 2019; PDLIM5: Yan et al., 2015; Liu et al., 2017; PDLIM7: Kales et al., 2014; Klein et al., 2018). This is particularly the case for PDLIM1 and PDLIM2, with their involvement in cancer being reviewed recently in Zhou et al. (2021) and Guo and Qu (2021). ALP/Enigma family members are also involved in hemostasis (Bozulic et al., 2007; Gupta et al., 2012; Krcmery et al., 2013; Urban et al., 2016), immune and inflammatory responses (Qu et al., 2012; Ono et al., 2015; Fu et al., 2019; Joyce et al., 2019; Yoo et al., 2019) and bone morphogenesis (Strohbach et al., 2008; Lin et al., 2010; Liu H et al., 2014), demonstrating the versatility of this protein family. The functional diversity of the ALP/Enigma proteins cannot be overstated, however, here we focus on their roles in muscle structure and development.

### ALP/Enigma family proteins in a muscle context

**FIGURE 2:**
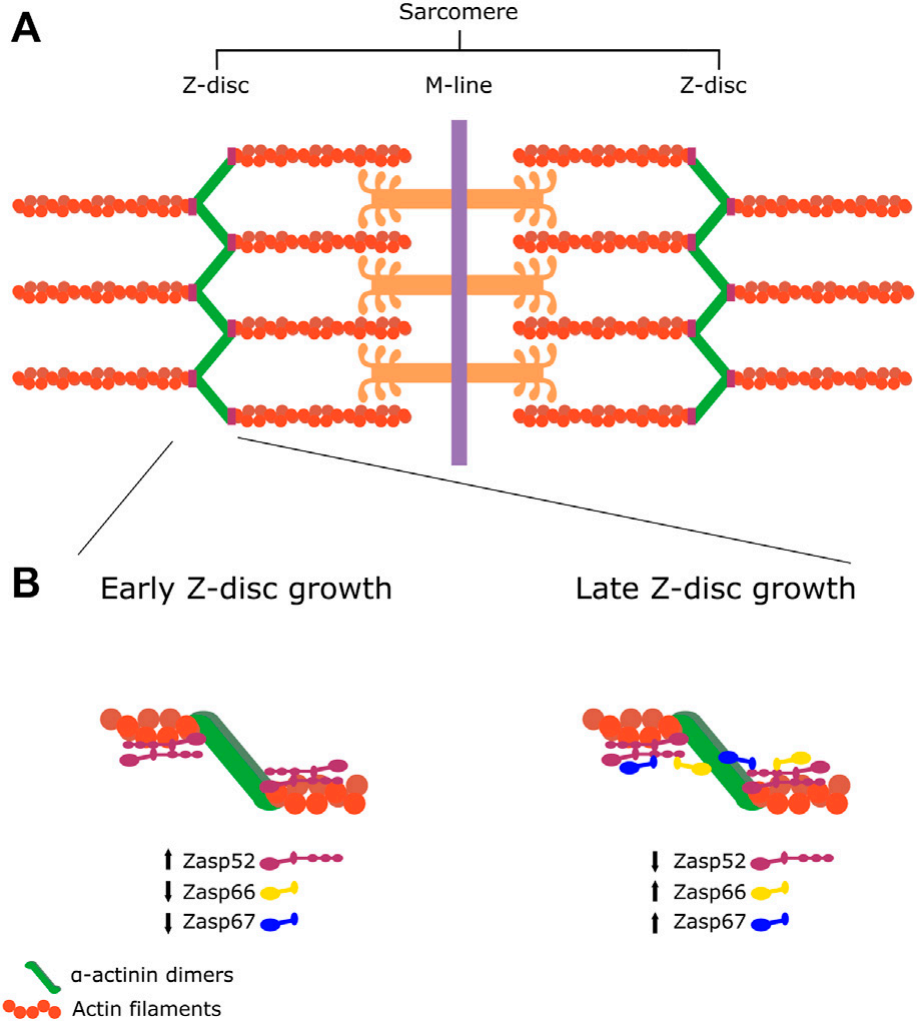
The sarcomere and Zasp52 during Z-disc growth **(A)** Structure of the sarcomere. The myosin thick filaments are linked at the M-line, and the actin thin filaments are anchored at the Z-disc. **(B)** Process uncovered by González-Morales et al. (2019b). During early Z-disc growth, the “growing” isoform (Zasp52) predominates and allows interaction with other Zasp proteins through the ZM/LIM interaction. During late Z-disc growth, the “blocking” isoforms with no LIM domains predominate (Zasp66 and Zasp67), which inhibits further growth of the Z-disc.

Muscles are made up of myofibrils, which are in turn made up of sarcomeres, the smallest contractile unit of the muscle cell (Figure 2A). The sarcomere contains a stereotypical structure; each unit is delineated by the Z-disc, a multiprotein structure which anchors the actin thin filaments. These are antiparallel to the myosin thick filaments, which are anchored at the M-line, and it is the sliding motion of the myosin thick filaments over the thin filaments that causes contraction. This contraction is heavily dependent on cytoskeletal dynamics, and therefore ALP/Enigma proteins are naturally implicated in this process.

Many of the ALP/Enigma proteins have critical roles in a muscle context. In a landmark study, Pashmforoush et al. demonstrated the role of ALP in the development of dilated cardiomyopathy through its interaction with *α*-actinin and stabilization of actin filament structure (Pashmforoush et al., 2001). Since then, many more studies have elucidated the role of ALP/Enigma members in muscle structure stability. In *C. elegans*, the sole ALP/Enigma protein ALP-1 stabilizes the actin filament structure (Han and Beckerle 2009). In regards to cardiac structural integrity, Enigma has been shown to cause valve dysfunction in both zebrafish and mouse heart models (Camarata et al., 2010; Krcmery et al., 2013).

ALP/Enigma proteins also promote differentiation of muscle cells, as well as cardiac remodelling. Recently for example, ALP and ENH have been shown to promote differentiation and proliferation of satellite cells in chicken skeletal muscle, with ALP being regulated by miRNAs (Yin et al., 2020; He et al., 2021). ENH is also involved in the regulation and remodelling of rodent hearts, with different splice variants leading to reduced or enhanced ventricular cardiomyocyte hypertrophy, in addition to being the target of miRNA silencing which promotes cardiomyocyte hypertrophy (Yamazaki et al., 2010; Bang et al., 2014). Moreover, Enigma has been found to be a target of the E3 ubiquitin ligase Nedd4-1 causing the development of muscle atrophy when degraded (D’Cruz et al., 2016). Members of the ALP/Enigma family are therefore involved in proliferation, differentiation, and maintenance of proper structure of muscle cells.

Perhaps the most prevalent and well researched ALP/Enigma protein in muscles is ZASP/Cypher. ZASP (Z-disc associated alternatively spliced protein) is a core component of the Z-disc. Ablation of ZASP (Cypher) in mouse heart muscles causes development of dilated cardiomyopathy with premature death (Zheng et al., 2008). ZASP has also been shown to be involved in myofibrillar myopathies and cardiomyopathies in humans (Arimura et al., 2009; von Nadelstadh et al., 2009; Lin et al., 2014). Therefore it is of great importance to understand binding partners, dynamics and the roles of ZASP in development. Recently, significant insights have been gained using *Drosophila melanogaster* as a model organism. The indirect flight muscles (IFM) of *Drosophila* have strong homology to human sarcomeres (reviewed in Lemke and Schnorrer 2017), thus making them an ideal model to study sarcomeric proteins. The next section will focus on recent advances undertaken in the *Drosophila* model system, and the implication this has for human muscle-related diseases.

### From insects to humans: Zasp52 and myofibril development

Zasp52, the *Drosophila* ZASP ortholog, is necessary for a myriad of processes in muscle cells. For starters, Zasp52 is essential for the maintenance and development of functional integrin adhesion sites, which is integral to cell spreading and the development of myotendinous junctions (Jani and Schöck, 2007). Moreover, Zasp52 has been shown to be required for integrin activation as well. Zasp52 activates integrins in dual color flow cytometric assays, and *Zasp52* mutants can be rescued by talin overexpression, the main activator of integrins (Bouaouina et al., 2012). More importantly, however, *in vivo* fluorescent recovery after photobleaching (FRAP) experiments have demonstrated that integrins are more mobile in *Zasp52* mutant embryos and talin mutants during later stages of *Drosophila* embryo development compared to their wild-type counterparts (Bouaouina et al., 2012), which is indicative of muscle detachment. Mature myofibrils are more static in their integrin adhesion sites in order to resist contractile forces (Yuan et al., 2010), and therefore this highlights the importance of Zasp52 as a regulator of cell signalling during contraction. Without functional integrin adhesion sites, muscle cells are not able to properly bind to the extracellular matrix, which is necessary to transmit contractility signals between neighbouring cells (reviewed in Sparrow and Schöck, 2009). Thus, Zasp52 appears to act as a key regulator of these signalling dynamics, although it is still unknown if Zasp52 acts directly on integrin or indirectly on Slik, which phosphorylates and thereby regulates talin (Katzemich et al., 2019).

In addition to being involved in adhesion sites, Zasp52 is also a core regulator of Z-disc structure, which delineates the sarcomere and anchors the actin thin filaments in the contractile apparatus. In *Drosophila* IFMs, Zasp52 localizes to Z-discs during early development and is necessary for the maintenance of the myofibril (Katzemich et al., 2013). In addition to Zasp52, there are two other ALP/Enigma proteins that were discovered in *Drosophila*, Zasp66 and Zasp67, which are also necessary for the assembly of myofibrils (González-Morales et al., 2019a). Double mutant knockdowns of Zasp52 with either Zasp66 or Zasp67 causes a more severe phenotype than Zasp52 alone or than the *α*-actinin null mutant phenotype, which is characterized by loss of Z-disc structure (Katzemich et al., 2013). This research suggests that each member of the ALP/Enigma family in *Drosophila* forms a multi-protein complex with *α*-actinin that is crucial for Z-disc formation during development. Recently, Zasp52 has also been shown to bind actin filaments directly (Liao et al., 2020). This may explain the incomplete rescue observed with a Zasp52 transgene containing only the *α*-actinin binding domain. By being able to bind to actin directly, Zasp52 would still be able to anchor Z-discs to the cytoskeleton and retain the structure of the contractile apparatus in some cases of muscle diseases. The region required for actin binding in Zasp52 includes the extended PDZ domain, and the ZM region that immediately follows, although it is still unknown if amino acids of the 26 amino acid ZM domain itself contribute to actin binding (Liao et al., 2020).

Apart from the PDZ and LIM domains, Zasp52 also contains the muscle-specific ZM domain. Recently, using the *Drosophila* IFM as a model, the importance of this domain has been elucidated (Figure 2B). One common property of sarcomeres is that in all organisms, they have an invariant length and width within one muscle type, and in vertebrates, even show invariance within the species. Until now, the mechanisms behind the determination of said width had not yet been established. The ZM domain may be a key factor in determining width of the sarcomeres. During muscle development, small structures called Z-bodies develop, and are the precursors of the mature sarcomere. These Z-bodies are complexes of *α*-actinin and associated proteins that grow in size and eventually dictate the final size of the sarcomere (Wang et al., 2005; González-Morales et al., 2019b). As mentioned earlier, Zasp isoforms make part of the Z-disc protein complex, and are critical for proper Z-disc development (Katzemich et al., 2013). All Zasp isoforms in *Drosophila* have a PDZ and ZM domain, while only Zasp52 contains additional LIM domains. Interestingly, temporal expression and localization of specific isoforms is what determines sarcomere growth. Zasp52, which contains LIM domains, has been shown to be a “growing” isoform of the Z-disc, while Zasp66 and Zasp67, lacking LIM domains, are “blocking isoforms.” The LIM domains and ZM domains interact, and it is this interaction that determines growth of the Z-disc. During the growth phase of the sarcomere, Zasp proteins with unbound LIM domains recruit additional Zasp proteins by the ZM domain. Each of the four LIM domains are able to interact with a ZM domain, which can increase the size of the sarcomere by recruiting additional growing isoforms. However, in the late phase, the ratio of growing to blocking isoforms changes, and more blocking isoforms lacking LIM domains begin binding to the already aggregated Zasp52 molecules. This terminates the growth of the sarcomere by impeding further growing isoform recruitment (González-Morales et al., 2019b).

Having shown the importance of Zasp in muscle cells and the sarcomere, we will now move on to what these discoveries mean for human myopathies, as well as other potential roles that ALP/Enigma proteins play in muscle-related diseases.

## Discussion

The pathology of myofibrillar myopathies originates in the Z-disc. Often in myofibrillar myopathies, symptom onset is caused by disintegration of the Z-disc, then the myofibrils, followed by an abnormal ectopic accumulation of multiple proteins (Selcen and Engel 2005). In Zasp-related myopathies, sometimes referred to as zaspopathies, prognosis follows a similar trajectory.

In contrast to *Drosophila* muscle, vertebrate skeletal muscle has high regenerative properties due to the presence of satellite cells, a group of quiescent muscle progenitors that proliferate, differentiate and fuse into pre-existing myofibers upon muscle injury (reviewed in Kang and Krauss 2010). These newly formed myofibers adopt the stereotypical sarcomeric structure, and undergo a similar molecular mechanism found in developmental myogenesis to reach their final state (reviewed in Bentzinger et al., 2012). This process requires a mechanism to set the diameter of the Z-disc, and therefore it is plausible that the process outlined in González-Morales et al. (2019b) is found in human myogenesis throughout the entire life cycle including muscle development and muscle regeneration caused by muscle damage and during hypertrophy. Interestingly, some Cypher (ZASP) and ENH (PDLIM5) single and double mutants show a similar phenotype than the one found in *Drosophila* sarcomeres in which the “blocking” isoform predominates, namely a smaller Z-disc diameter (Zhou et al., 2001; Cheng et al., 2010; Mu et al., 2015). In humans, there are ZASP isoforms that lack all LIM domains and are comprised solely of the PDZ and ZM domains, as well as the ALP subfamily in which the proteins only possess one LIM domain (Cheng et al., 2011; reviewed in Zheng et al., 2009). These proteins could represent the “blocking” isoforms in the *Drosophila* Z-disc diameter model which would function to end Z-disc growth at the specified diameter. Interestingly, in mouse models, when the larger form of ZASP containing all three LIM domains is knocked out, neonatal lethality and Z-disc perturbations are observed, while knock-out of the shorter ZASP isoform containing no LIM domains conversely has no phenotype (Cheng et al., 2011). Moreover, ENH shows a similar pattern of isoform transition from a LIM-containing to LIM-less splice variant during mouse heart development (Yamazaki et al., 2010; Ito et al., 2012). In addition, there are many ZM domain as well as LIM domain mutations found in zaspopathies (Vatta et al., 2003; Selcen and Engel 2005; Theis et al., 2006) which lends further credence to this hypothesis.

Recently, SNPs in PDLIM3 and PDLIM5 have been implicated in the development of idiopathic dilated cardiomyopathy (Wang et al., 2019). Additionally, loss of function variants in PDLIM3 have been correlated with atrial fibrillation, a common cardiac arrhythmia (Vad et al., 2020), as well as hypertrophic cardiomyopathy (Lopes et al., 2015). These recent findings highlight the growing relevance of ALP/Enigma proteins during muscle and heart development, and possible implications for human disease. In mammals, the ALP/Enigma proteins have been proposed to have redundant roles because of their shared domains (Jo et al., 2001; Mu et al., 2015; reviewed in Zheng et al., 2009). However, if mutations are compounded, the redundancy of the proteins may not suffice for proper muscle function. Fichna et al. (2018) have proposed different possible inheritance modes for myofibrillar myopathies in their review, two of which involve the accumulation of minor or benign variants which can in conjunction compound and lead to the development of disease. There is already some evidence to support this with the ALP/Enigma family. Recently, PDLIM5 has been suggested to be a disease modifier in familial DCM cases caused by mutations in the *TTN* gene (Verdonschot et al., 2019). Additionally, lower mRNA expression of PDLIM5 was shown to be matched with an increase in mRNA expression of ZASP, one of its interactors in the Z-disc and member of the ALP/Enigma family, which validates previous *PDLIM5*-knockout mouse model data (Cheng et al., 2010). Therefore, it is entirely possible that this process could be relevant for ALP/Enigma proteins in a muscle context. A patient with a benign or minor variation in ZASP may be more susceptible to developing a myopathy if there are other mutations in redundant ALP/Enigma family proteins, such as PDLIM3 or PDLIM5, which are now no longer able to provide the structural integrity of the Z-disc.

The ALP/Enigma proteins are a diverse group of adaptor proteins involved in numerous and vastly different cellular processes. In this review, we highlighted the evolution of their protein structure. It is from the complexity of their domains that their versatility is conferred, which enables them to bind a myriad of different proteins. We also covered recent advances in *Drosophila*, showcasing the potential implications for muscle disorders. The mounting research in recent years on the ALP/Enigma family proteins is uncovering the families’ relevance for muscle-related diseases and possible therapeutic targets in a clinical setting. More research will need to be carried out in order to elucidate therapeutic avenues, as well as examining the possible genetic interactions between multiple members of the family in a myopathic context. Of particular interest should be future studies investigating if sarcomere diameter is controlled similarly in vertebrates as in insects and what this means for human myopathies.
